# HDC downregulation induced by chronic stress promotes ovarian cancer progression via the IL-6/STAT3/S100A9 pathway

**DOI:** 10.3389/fphar.2024.1485885

**Published:** 2024-12-10

**Authors:** Zhicong Chen, Jinming Cao, Zhijun Xiao, Zhen Yang, Yuanchi Cheng, Jingjing Duan, Ting Zhou, Feng Xu

**Affiliations:** ^1^ Fengxian Hospital, School of Pharmaceutical Sciences, Southern Medical University, Shanghai, China; ^2^ Sixth People’s Hospital South Campus, Shanghai Jiaotong University, Shanghai, China

**Keywords:** histidine decarboxylase, histamine, depression, ovarian cancer, chronic stress

## Abstract

**Objective:**

This study aimed to investigate the underlying mechanism of chronic stress promoting ovarian cancer growth comorbid with depression and evaluate the potential role of histamine (HIS) in treating this comorbidity.

**Methods:**

Chronic unpredictable mild stress (CUMS) was used to establish a comorbid mouse model of ovarian cancer and depression. The behavioral phenotypes were assessed using the sucrose preference test (SPT), tail suspension test (TST), forced swimming test (FST), and open field test (OFT). Ovarian cancer growth was monitored by tracking the tumor volume and weight. Histidine decarboxylase (HDC) expression in the tumor tissue was analyzed using Western blot and qRT-PCR techniques. The serum levels of inflammatory factors (IL-6 and IL-17A), stress hormones (norepinephrine, NE and cortisol, and COR), histamine, and 5-hydroxytryptamine (5-HT) were detected by enzyme-linked immunosorbent assay (ELISA). *In vitro* experiments were conducted to explore the direct impacts of stress hormones on A2780 and ES-2 ovarian cancer cell lines, as well as the modulation of these effects by histamine. HDC knockdown and overexpression approaches were used to study its regulatory role in the IL-6/STAT3/S100A9 signaling pathway.

**Results:**

Chronic stress not only induced depressive behaviors but also accelerated ovarian cancer growth in mice by downregulating HDC expression in tumors, whereas exogenous HIS treatment alleviated depressive symptoms, suppressed cancer growth, and countered the decreased levels of HIS and increased levels of IL-6, IL-17A, NE, COR, and 5-HT induced by CUMS. Furthermore, HIS positively modulated the immune response by increasing the populations of CD3^+^T and CD8^+^ T cells and reducing IL-17A secretion. *In vitro* experiments revealed that stress hormones downregulated HDC expression, consequently promoting cancer cell proliferation, migration, and invasion via the IL-6/STAT3/S100A9 pathway. Knockdown of HDC activated this pathway, whereas HDC overexpression inhibited its activation.

**Conclusion:**

Chronic stress leads to the downregulation of HDC expression, thereby facilitating the progression of ovarian cancer through the IL-6/STAT3/S100A9 pathway. HIS might serve as a potential molecule for treating the comorbidities of ovarian cancer and depression.

## Introduction

Ovarian cancer is the third-most common gynecologic malignancy but accounts for the highest mortality rate among gynecological cancers worldwide (Kuroki and Guntupalli, 2020). According to the global cancer statistics published by the World Health Organization (WHO), there were 324,398 new cases of ovarian cancer and 206,839 deaths worldwide in 2022 (Bray et al., 2024).

Chronic stress is a long-term psychological and physiological exposure process triggered by mainly negative environmental and/or psychosocial factors, which disrupts the internal homeostasis of the body (He et al., 2024). Chronic stress contributes to the development and/or maintenance of mental health problems and results in depression (Lupien et al., 2018; Hammen, 2005). The chronic unpredictable mild stress (CUMS) animal model, a widely used and reliable rodent model for depression, can effectively mimic human depression and is used extensively in the research on depression (Antoniuk et al., 2019; Nollet, 2021; Monteggia et al., 2014).

Many clinical investigations show that cancer patients suffer from a high incidence of major depressive disorder. Ovarian cancer patients report an even higher incidence of depression ranging from 17% to 21% (Lutgendorf and Andersen, 2015; Price et al., 2010). Comorbid depression in cancer patients may promote cancer incidence, progression, and metastasis, resulting in higher cancer-related mortality rates (Powell et al., 2013; Zhao et al., 2015; He et al., 2024). There is an interplay between chronic stress, depression, and cancer, which emphasizes the significant connection between psychological and physiological factors in cancer progression and management.

Histidine decarboxylase (HDC) is a key enzyme involved in endogenous histamine (HIS) synthesis in the human body, which catalyzes the decarboxylation of L-histidine into HIS. Both HDC and HIS have pivotal roles in nervous system functioning, such as regulating sleep–wakefulness, working memory, and antidepressant-like effects (Thakkar, 2011; Nakamura et al., 2022; Costa et al., 2018). In our previous studies, we found that HDC mRNA was significantly decreased both in depression patients and in chronic stress model mice (Zhou et al., 2021). HDC and HIS are involved with the formation, progression, and metastasis of various tumors (Yang et al., 2011; Froldi et al., 2019; Ahn et al., 2015). Depression and ovarian cancer have a mutually reinforcing relationship; however, there is lack of research on exploring the HDC expression profile in comorbid depression in ovarian cancer (Brown et al., 2024; Hathaway et al., 2023; Logue et al., 2020).

The IL-6/STAT3/S100A9 signaling pathway drives the proliferation, survival, invasion, and metastasis of tumor cells; meanwhile, it strongly inhibits the antitumor immune response (Johnson et al., 2018; Böttcher et al., 2022). This study aimed to investigate the impact of chronic stress on HDC expression in ovarian cancer progression and the effect of HIS on the IL-6/STAT3/S100A9 pathway.

## Materials and methods

### Animal

The study protocol was approved by the Institutional Experimental Animal Ethics Committee of Fengxian Hospital, Southern Medical University (No. 2022-0334). Female C57BL/6 mice (15–18 g, aged 5 weeks) were obtained from Guangdong DK Pharmaceutical Co. Ltd. (License No: SCXK 2022-0060). The mice were housed in cages collectively for the control group and individually for the CUMS group at controlled temperature. A week-long acclimatization was provided, during which the mice had *ad libitum* access to food and water.

**FIGURE 1:**
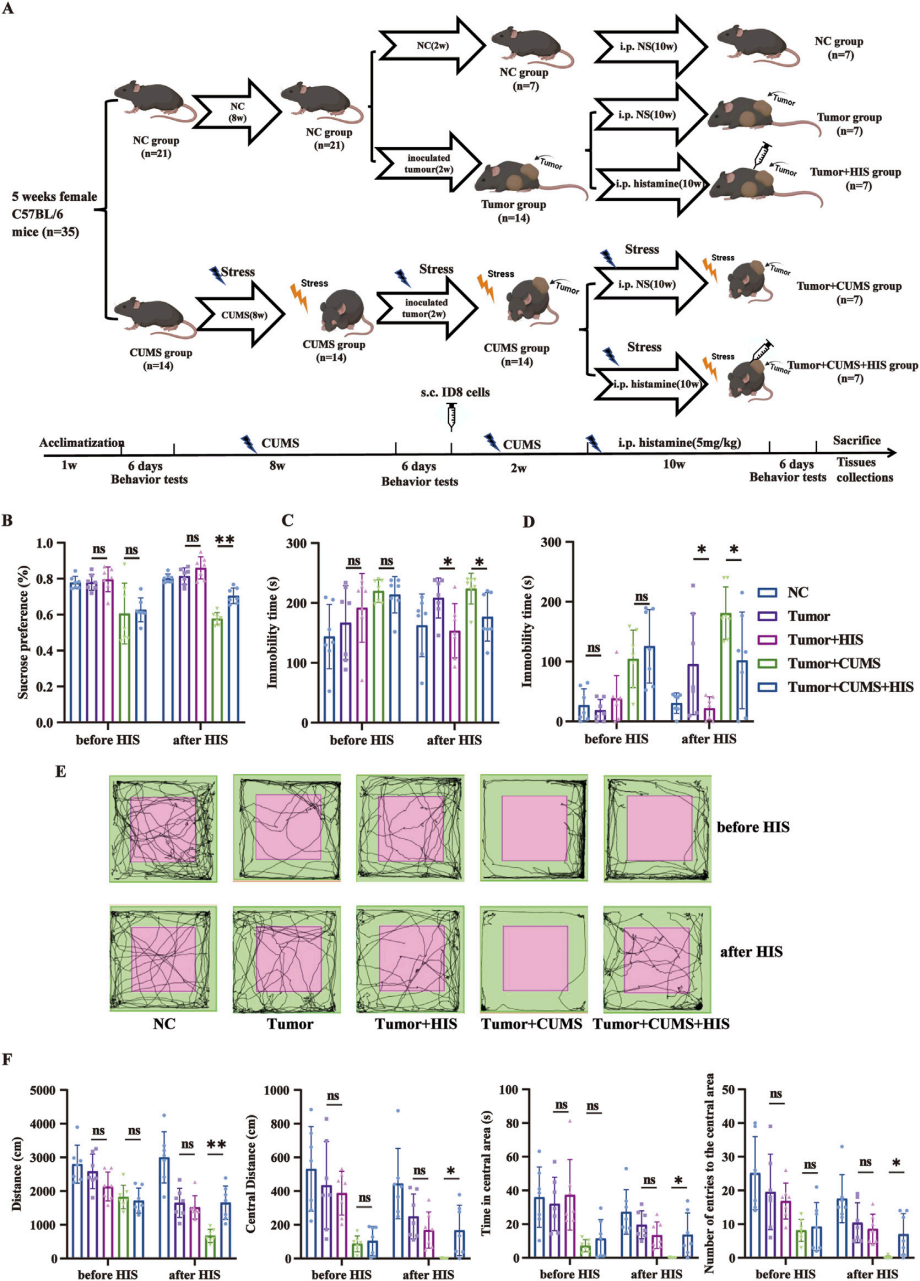
HIS treatment improved depression-like behaviors exacerbated by exposure to CUMS. **(A)** Scheme of the animal experimental design. Thirty-five female C57BL/6 mice were randomly divided into two groups: the normal control (NC) group (n = 21) and the CUMS group (n = 14). After initial behavior tests, the CUMS group was exposed to CUMS for 8 weeks, and then all mice were reassessed through a series of behavior tests. Subsequently, the NC group was further randomly divided into three subgroups: the NC subgroup, the tumor subgroup, and the tumor + HIS subgroup (n = 7 each). Meanwhile, the CUMS group was randomly divided into two subgroups: the tumor + CUMS subgroup and the tumor + CUMS + HIS subgroup (n = 7 each). Each subgroup, except the NC subgroup, received an injection of 1 × 10^6^ ID8 mouse ovarian cancer cells into the posterior right limb. Two weeks after inoculation, mice in the tumor + HIS and tumor + CUMS + HIS subgroups were treated with an intraperitoneal injection of 5 mg/kg HIS, twice weekly, for 10 weeks. All mice were reassessed through behavior tests before being sacrificed. **(B)** Sucrose preference in the SPT. **(C)** Time of immobility in the TST. **(D)** Time of immobility in the FST. **(E)** Activity tracks in the OFT. **(F)** Locomotion activity scores in the OFT. Data are shown as mean ± SD. *p < 0.05, **p < 0.01 (n = 7 each).

The scheme of the animal experimental design is shown in Figure 1A. At the endpoint, all mice were euthanized, and then blood serum, tumors, and spleens were collected. Tumors were photographed and weighed, with a portion of the tumor fixed in formalin for histopathology staining, and the remaining tumor tissue was used for Western blot and qRT-PCR experiments. The spleens were processed into single-cell suspensions for flow cytometry analysis.

### CUMS procedure

The CUMS procedure was performed according to the protocol (Nollet, 2021), with a slight modification. Mice in the CUMS group were exposed to two different stressors daily. The detail of the specific interventions performed is shown in Supplementary Table S1.

### Behavioral assessments

Behavioral assessments were conducted consistently in 6 days, including the sucrose preference test (SPT), tail suspension test (TST), forced swimming test (FST), and open field test (OFT). Each behavioral evaluation was conducted with a 24-h interval between tests. The detail of behavioral testing is shown in our previous research (Li et al., 2023).

### Subcutaneous heterotopic transplantation ID8 model

The ID8 cell line was obtained from the Cell Bank of the Chinese Academy of Sciences (Shanghai, China) and maintained in the laboratory. Cells were cultured in DMEM high glucose medium (Gibco, California, United States) supplemented with 10% fetal bovine serum (FBS) (Invitrogen, California, United States), penicillin (10 U/mL), and streptomycin (100 mg/mL). Mice were subcutaneously injected with 1 × 10^6^/mL ID8 cells in 100 µL of phosphate-buffered saline (PBS) to establish a subcutaneous heterotopic transplantation tumor model. Tumor volume was measured every week.

### Immunohistochemistry (IHC)

Tumor tissues of mice were fixed in 10% (v/v) formaldehyde in PBS, embedded in paraffin, cut into 4-μm sections, and used for immunohistochemistry (IHC) staining with antibodies against Ki67 (1:1,000, Abcam, United Kingdom) and HDC polyclonal antibody (Immunoway Biotechnology, United States). The representative images were acquired under a light microscope (Olympus IX73, Tokyo, Japan). Ki67 index was calculated as the number of immunopositive cells × 100% divided by the total number of cells in seven random fields at ×400 magnification.

### Blood sample analysis with enzyme-linked immunosorbent assay (ELISA)

HIS (Elabscience, China), NE (Fine Test, China), COR (Fine Test, China), IL-6 (Fine Test, China), IL-17A (Elabscience, China), and 5-HT (Fine Test, China) in serum and in cell supernatant was analyzed using ELISA kits following the manufacturer’s instructions.

### Hematoxylin and eosin (H&E) staining

The tumor tissues were fixed in 4% paraformaldehyde and embedded in paraffin. The sliced sections were dewaxed using solvents, stained with hematoxylin and eosin, and photographed under a microscope.

### Protein expression analysis by Western blotting

The expressions of HDC, STAT3, p-STAT3, S100A9, and GAPDH proteins were analyzed by Western blotting. The primary antibodies used include HDC polyclonal antibody (Immunoway Biotechnology, United States), Stat3 (124H6) antibody (Cell Signaling, Massachusetts, United States), Phospho-Stat3 (Tyr705) (D3A7) XP^®^ antibody (Cell Signaling, Massachusetts, United States), S100A9 antibody (Invitrogen California, Texas, United States), and GAPDH (ABclonal, China). The band density was analyzed using a gel imaging system and compared with an internal control.

### mRNA expression analysis by qRT-PCR

Total RNA was extracted with TRIzol (Invitrogen™, Carlsbad, United States). The extracted RNA was reverse-transcribed to complementary DNA (cDNA) using an Evo M-MLV RT Mix Kit (ACCURATE BIOTECHNOLOGY CO., LTD., Changsha, China). For qRT-PCR, a SYBR Green Premix Pro Taq HS qPCR Kit (ACCURATE BIOTECHNOLOGY CO., LTD., Changsha, China) was utilized following the manufacturer’s protocol. Relative gene expression was calculated using the 2^−ΔΔCT^ method. The list of primers is shown in Supplementary Table S2.

### Flow cytometric analysis of T cell

Mouse spleen single-cell suspensions were stained with specific antibody for 20 min, including PE anti-mouse CD4 (Biolegend, California, United States), FITC anti-mouse CD8a (Biolegend, California, United States), and PerCP/Cyanine5.5 anti-mouse CD3 (Biolegend, California, United States) and then were analyzed using a flow cytometer (Canto; BD Biosciences, Shanghai, China) equipped with Cell-Quest software (BD Biosciences).

### Cell lines, culture conditions, and cell transfection

A2780 and ES-2 cell lines were obtained from the Cell Bank of the Chinese Academy of Sciences (Shanghai, China) and maintained in the laboratory. A2780 cells were cultured in DMEM high glucose medium (Gibco, California, United States) supplemented with 10% FBS (Invitrogen, California, United States), penicillin (10 U/mL), and streptomycin (100 mg/mL). ES-2 cells were cultured in RPMI 1640 medium (Gibco, California, United States) supplemented with 10% FBS (Invitrogen, California, United States), penicillin (10 U/mL), and streptomycin (100 mg/mL). All cell lines were cultured in a humidified atmosphere of 5% CO_2_ at 37°C.

### Drug treatment

For the *in vitro* cell test, four groups were set up with different treatment types: the normal control (NC) group was cultured in de-hormone medium; NE + COR group with 10 μM NE and 1.5 μM COR (Sigma-Aldrich, Saint Louis, United States) in the medium; NE + COR + HIS group with 10 μM NE, 1.5 μM COR, and 10 μM HIS (APExBIO, Houston, United States) in the medium; and BHOA group with 1 mM BHOA (APExBIO, Houston, United States) in the medium.

### Construction of HDC knockdown and overexpression cell lines

HDC knockdown or overexpression cell lines were generated with transiently transfected cells with HDC siRNA or HDC overexpression plasmid, respectively (Hanbio Tech, Shanghai, China). The vectors of OE-HDC were pcDNA3.1-CMV-MCS-3flag-EF1-ZsGreen-T2A-Puro. Specific HDC siRNA and HDC overexpression plasmid were transfected using Lipofectamine 3000 (Thermo Fisher Scientific, Massachusetts, United States) for 48 h. The transfection efficiency was observed through a fluorescence microscope, and HDC expression was determined through Western blotting analysis. Cells transfected with non-targeting siRNA (siNC group) and empty plasmid vectors (vector group) were used as controls. The transfected cells were then utilized to establish the siHDC group (HDC knockdown) and the OE-HDC group (HDC overexpression). Details of the siRNA and overexpression plasmid sequences used are listed in Supplementary Table S3.

### Cell Counting Kit-8 (CCK-8) assay

Cells (3,000 per well) were seeded into 96-well plates. Ten microliters of Cell Counting Kit-8 solution (NCM Biotech, Suzhou, China) was added to each well after the cells were incubated at 37°C for 24–120 h. The optical density (OD) was then measured at 450 nm. Cell viability (%) = [(OD value of experimental group - OD value of blank control group)/(OD value of control group - OD value of blank control group)] × 100%.

### Clone formation assay

Cells seeded in 6-well plates (1 × 10^2^/well) were serum-starved and cultured in hormone-depleted medium with various treatments for 10–14 days (medium changed every three days). Cells were fixed as the number of cells in a single clone exceeded 50, stained with crystal violet, dried, and photographed.

### Scratch healing assay

Cells were seeded in 6-well plates (1 × 10^6^/well) and serum-starved for 12 h. Wounds were scratched using sterile tips, followed by washing in PBS. Cell migration was observed and photographed at 0, 24, and 48 h, and the mobility was calculated.

### Cell migration assay

Cells were resuspended in serum-free medium at 2.5 × 10^5^ cells/mL. The transwell chamber was placed in a 24-well plate, with 600 µL of 10% FBS and treatment solution (10 µM NE + 1.5 µM COR or 10 µM NE + 1.5 µM COR + 10 µM HIS) in the lower chamber and 200 µL of the cell suspension in the upper chamber. After 12–24 h of incubation at 37°C, cells on the underside of the membrane were fixed with 4% paraformaldehyde for 30 min and stained with 0.1% crystal violet for 30 min. Cells residing at the bottom of the chamber were gently removed, and the chamber was washed. After drying, six random fields were photographed under a microscope (100×) per chamber, and the number of migrated cells was counted and recorded.

### Cell invasion assay

Cells were coated with Matrigel (Corning, New York, United States) 4 h in advance, and the other steps were the same as for the cell migration assay.

### Statistical analysis

All the data are expressed as mean ± standard deviation (SD). The GraphPad Prism 9.5 software package was used for statistical analysis and visualization. Three independent experiments were carried out with similar results in qRT-PCR, Western blotting, ELISA, clone formation assay, scratch healing assay, cell migration assay, and cell invasion assay. Data were analyzed with an unpaired Student’s t-test and one-way analysis of variance. *p*-value <0.05 was considered statistically significant.

## Results

### CUMS induced depression-like behaviors

To explore the effects of CUMS on depression-like behaviors in mice, the SPT, TST, FST, and OFT were performed before and after CUMS to verify the depression-like behaviors (Supplementary Figure S1). Specifically, the sucrose preference was significantly lower in the CUMS group in the SPT compared with the normal control (NC) group (*p* < 0.01). The immobility time in the CUMS group was increased significantly in the TST and FST compared with that in the NC group (*p* < 0.01). Mice exposed to CUMS tended to move less in the OFT, with significant reductions in total distance, distance in central area, time in central area, and number of entries to the central area compared with the NC mice (*p* < 0.01, respectively). These results confirm that the depression model was successfully built.

### HIS treatment alleviated depression-like behaviors induced by CUMS

To investigate the impact of HIS treatment on depression-like behaviors in mice, we compared the behavioral outcomes of mice before and after HIS treatment. We found that HIS treatment effectively alleviated depression-like behaviors induced by CUMS in the ovarian cancer comorbid depression mouse model. Specifically, HIS increased sucrose preference in the SPT (Figure 1B), decreased immobility time in the TST (Figure 1C) and FST (Figure 1D), and decreased total distance, distance in central area, time in central area, and number of entries to the central area in the OFT (Figures 1E, F) compared with the tumor + CUMS group.

### CUMS promoted ovarian cancer progression, while HIS inhibited CUMS-induced tumor growth *in vivo*

**FIGURE 2:**
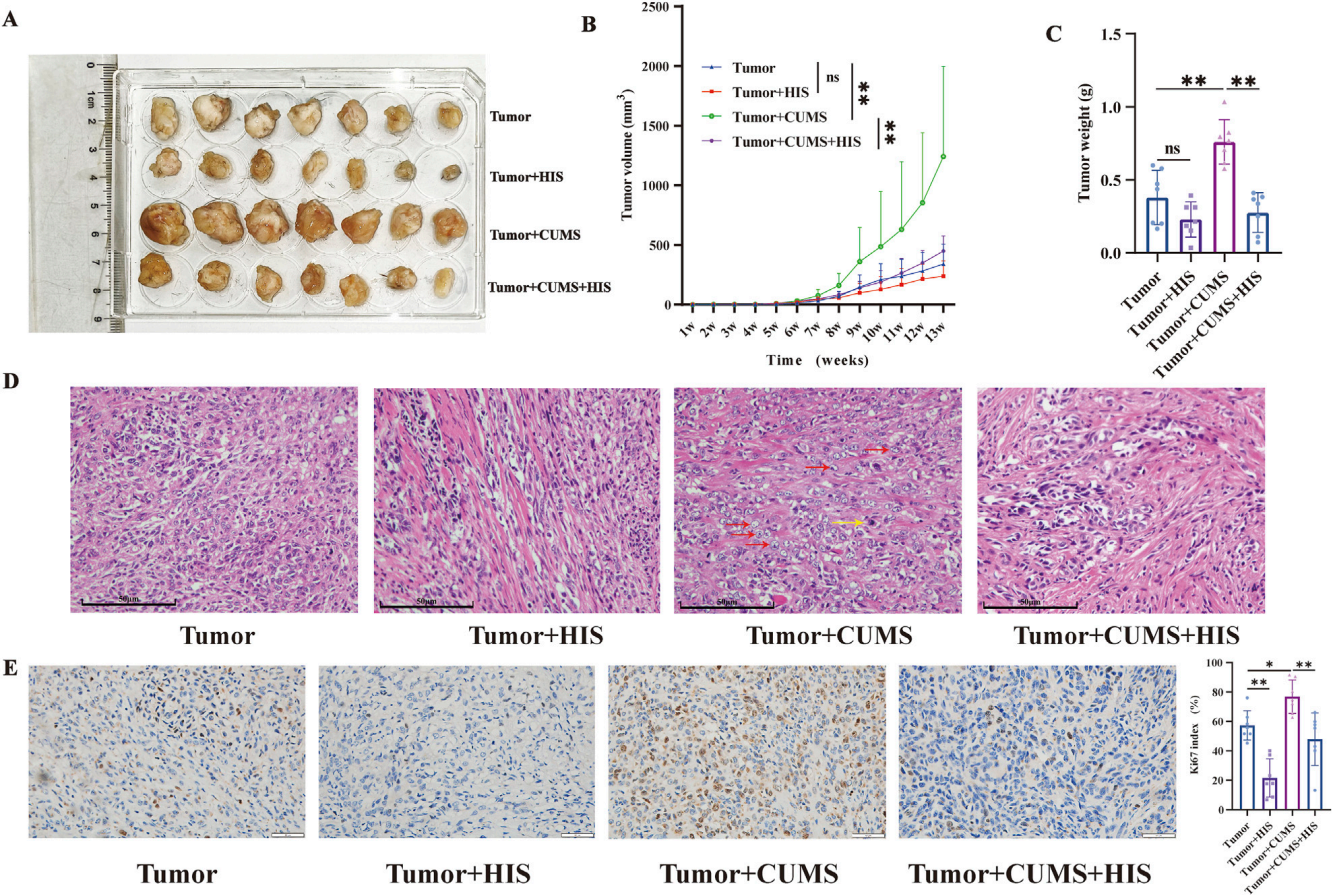
CUMS promoted ovarian cancer progression, while HIS inhibited CUMS-induced tumor growth. **(A)** Image of tumors in different groups. **(B)** Tumor volume change over time after tumor inoculation. **(C)** Tumor weight. **(D)** H&E straining of the tumor tissue. Prominent nucleoli (red arrows) and pathological nuclear division (yellow arrow). Scale bars = 50 μm. **(E)** Representative Ki67 staining in tumor tissues of different groups. Scale bars = 50 μm. Data are shown as mean ± SD. *p < 0.05; **p < 0.01. (n = 7 each).

To investigate the effects of chronic stress and histamine treatment on tumor progression *in vivo*, we measured diverse tumor features. The images of tumors in different groups are shown in Figure 2A. Compared with the tumor group, the tumor volume and weight of the tumor + CUMS group were increased significantly (*p* < 0.01 respectively), while the tumor volume and weight of the tumor + HIS group were decreased, with no significant difference. Compared with the tumor + CUMS group, the tumor volume (*p* < 0.05) and weight (*p* < 0.01) of the tumor + CUMS + HIS group decreased significantly (Figures 2B, C). Additionally, H&E staining of the tumor tissue showed that compared with the tumor group, the tumor + CUMS group exhibited an increase in the nucleus-to-cytoplasm ratio, distinct nucleoli, and pathological nuclear division. In contrast, compared with the tumor + CUMS group, the tumor + CUMS + HIS group showed a decrease in tumor cells and an increase in stroma (Figure 2D). Histopathological examination suggested that CUMS enhanced the Ki67 index, a marker of cell proliferation, while HIS treatment reduced the Ki67 index (*p* < 0.01, respectively, Figure 2E).

### Exogenous HIS treatment reversed the CUMS-induced alterations in mice serum *in vivo*

**FIGURE 3:**
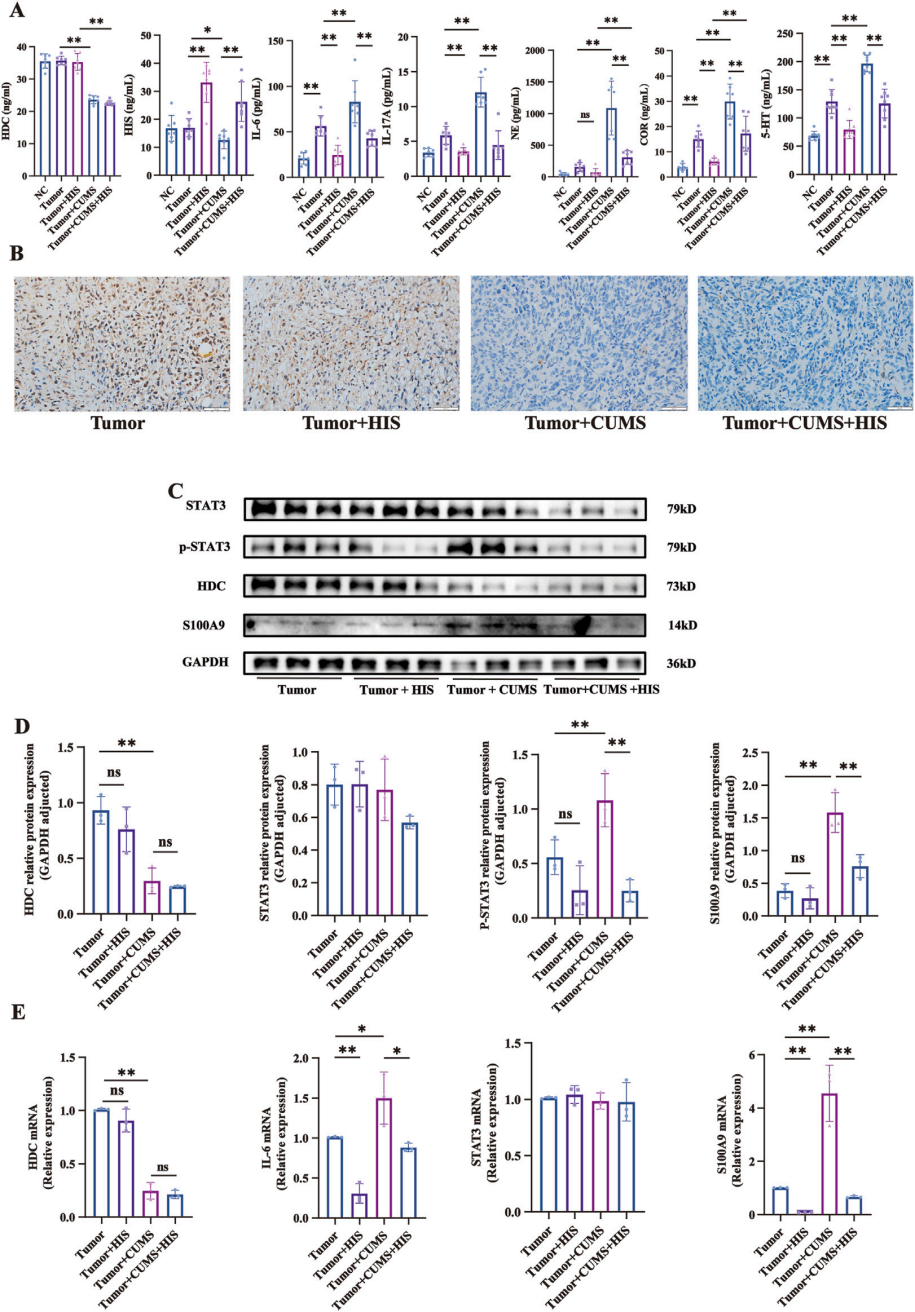
Changes of serum components, protein, and mRNA levels induced by CUMS and HIS treatment *in vivo*. **(A)** Levels of HDC, HIS, IL-6, IL-17, NE, COR, and 5-HT in serum of mice detected by ELISA (n = 7 each). **(B)** HDC expression was evaluated by immunohistochemistry. Scale bar = 50 μm. (n = 7 each). **(C)** Expressions of HDC, STAT3, p-STAT3, and S100A9 proteins in tumor of mice detected by Western blot. **(D)** Gray values of relative protein expression adjusted by GAPDH. **(E)** mRNA relative expression of HDC, IL-6, STAT3, and S100A9 in tumors of mice detected by qRT-PCR. Data are shown as mean ± SD. *p < 0.05, **p < 0.01.

In exploring the interplay between chronic stress and HIS in tumor progression, we observed that CUMS profoundly altered key biochemical markers in mice serum. Compared with the tumor group, CUMS significantly decreased HDC and HIS levels and increased IL-6, IL-17A, NE, COR, and 5-HT levels. Exogenous HIS treatment significantly increased HIS levels and decreased IL-6, IL-17A, COR, and 5-HT levels, with no significant effect on NE. Our findings indicated that CUMS changed the levels of HDC, HIS, inflammatory factors, stress hormones, and 5-HT, and HIS treatment reversed these changes, except for HDC (Figure 3A).

### CUMS downregulated HDC protein expression and activated the IL-6/STAT3/S100A9 pathway, and HIS reversed the over-activation of the IL-6/STAT3/S100A9 pathway induced by CUMS *in vivo*

Given that chronic stress promoted tumor growth while histamine treatment alleviated this effect, yet the regulatory mechanisms of HDC remain unclear, so we analyzed mouse tumor gene expression using immunohistochemistry, Western blot, and qRT-PCR. Immunohistochemical staining experiments specifically indicated that CUMS significantly reduced the expression of HDC in mouse tumors (Figure 3B).

Western blot analysis indicated that compared with the tumor group, the expression of HDC protein in the tumor + CUMS group was significantly decreased (*p* < 0.01). In contrast, the levels of p-STAT3 and S100A9 proteins were significantly increased (*p* < 0.01), while there was no significant difference in STAT3 protein expression. When comparing the tumor + CUMS group with the tumor + CUMS + HIS group, the expressions of p-STAT3 (*p* < 0.01) and S100A9 (*p* < 0.01) were significantly decreased in the tumor + CUMS + HIS group, with no significant differences in HDC and STAT3 proteins (Figures 3C, D). Similar results were observed for the changes in the mRNA expression levels (Figure 3E). The above results indicate that CUMS-induced downregulation of HDC leads to increased secretion of IL-6, phosphorylation of STAT3, and expression of S100A9, thereby activating the IL-6/STAT3/S100A9 pathway, whereas HIS effectively reversed the over-activation of this pathway induced by CUMS.

### HIS enhanced cellular immunity in tumor-bearing mice *in vivo*

**FIGURE 4:**
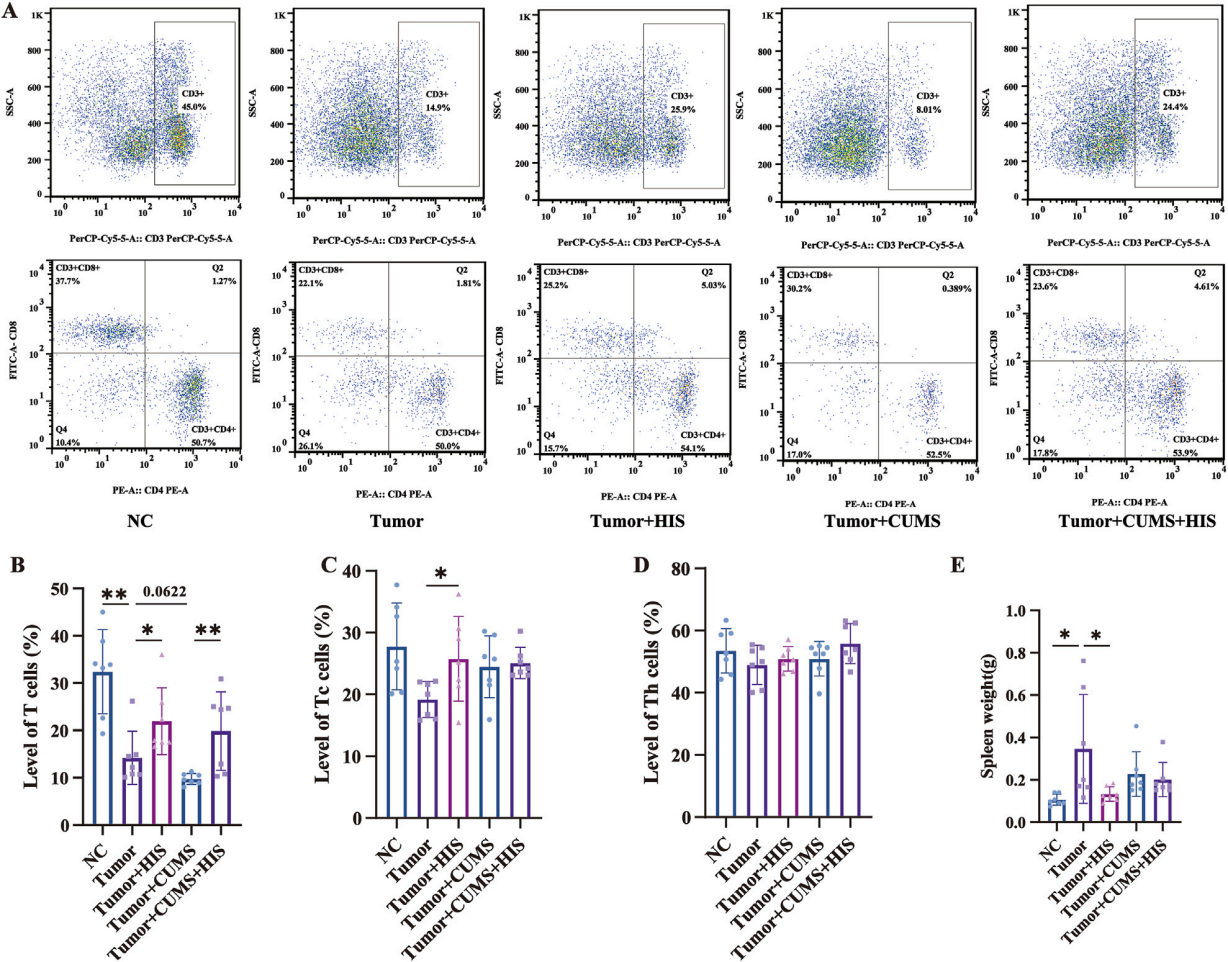
Tumor inoculation decreased T-cell proportion and increased spleen weight, while HIS increased T-cell proportion and decreased spleen weight. The percentages of CD3^+^ T **(A, B)**, CD3^+^ CD8^+^ Tc **(A, C)**, and CD3^+^ CD4^+^ Th **(A, D)** cells in the spleen of mice detected by flow cytometry. **(E)** Weight of spleen. Data are shown as mean ± SD. *p < 0.05; **p < 0.01 (n = 7 each).

To explore the effects of CUMS and HIS treatment on mouse immunity, we performed flow cytometry to measure the proportions of various immune cells (Figure 4A). Specifically, the proportion of CD3^+^ T cells (Figure 4B) was significantly reduced (*p* < 0.01) in the tumor group than in the NC group. HIS treatment significantly increased the levels of CD3^+^CD8^+^ Tc cells (Figure 4C) and CD3^+^ T cells compared with the tumor group (*p* < 0.05). Additionally, the proportion of CD3^+^ T cells in the tumor + CUMS + HIS group was significantly higher than that in the tumor + CUMS group (*p* < 0.05). The proportion of CD3^+^CD4^+^ Th (Figure 4D) cells remained stable. Furthermore, compared with the NC group, the spleen weight of mice in the tumor group was higher (*p* < 0.05), while HIS treatment reduced the spleen weight of mice in the tumor + HIS group compared with the tumor group (Figure 4E).

### Stress hormones promoted cell proliferation, migration, and invasion, while HIS treatment inhibited it *in vitro*

**FIGURE 5:**
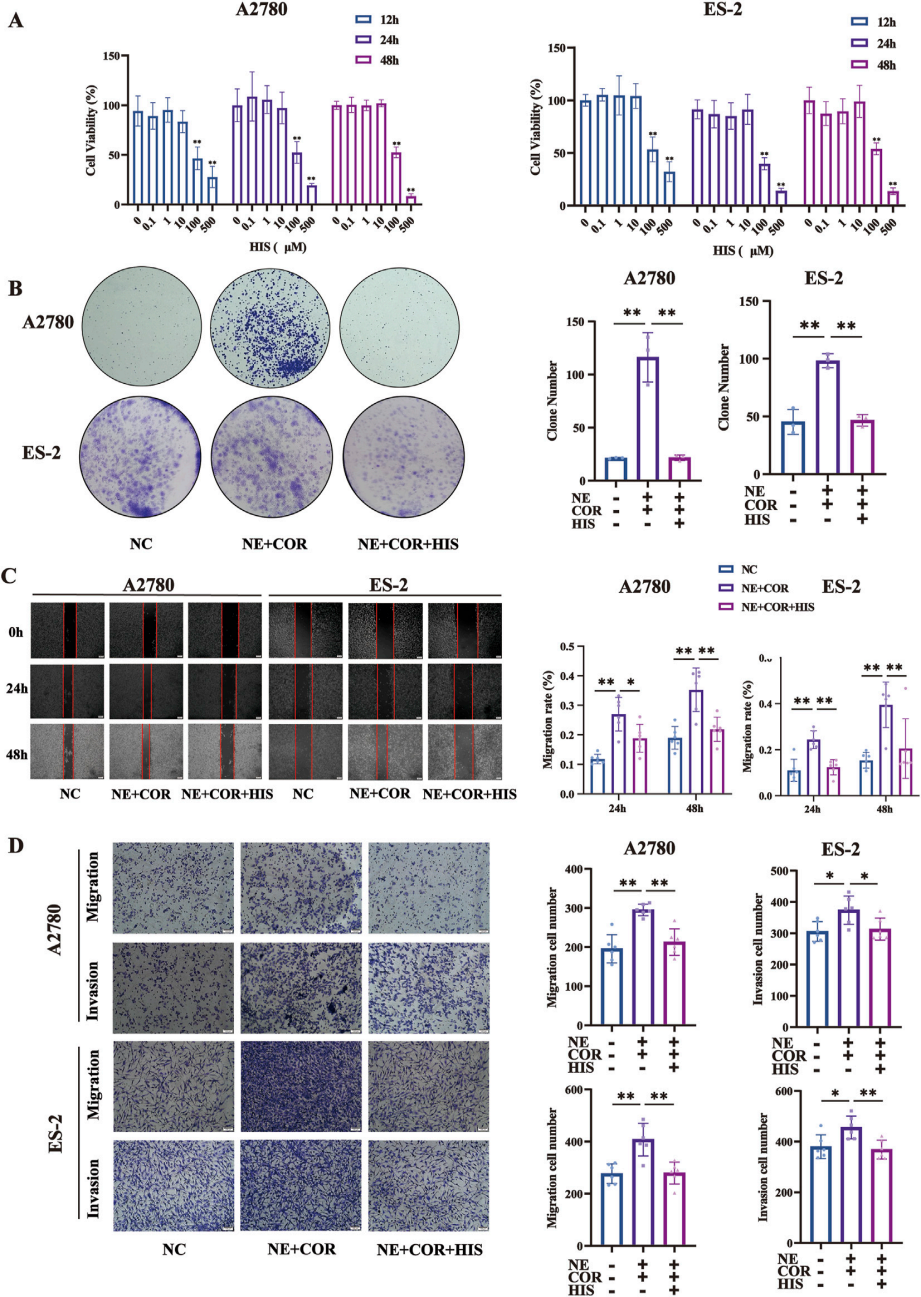
HIS treatment inhibited cell proliferation, migration, and invasion induced by stress hormones. **(A)** CCK-8 assay in A2780 and ES-2 cell lines treated with different concentration of HIS, * compared with 0 μM. **(B)** Colony formation assay in A2780 and ES-2. **(C)** Cell wound healing assay (100×) in A2780 and ES-2 cells. Scale bar = 200 μm. **(D)** Cell migration and invasion (100×) in A2780 and ES-2 cells. Scale bar = 100 μm. Data are shown as mean ± SD. *p < 0.05; **p < 0.01.

To further validate the impact of chronic stress on ovarian cancer progression, we employed an *in vitro* approach by treating A2780 and ES-2 cell lines with stress hormones and HIS. CCK-8 analysis showed that 100 μM and 500 µM HIS suppressed A2780 and ES-2 cell proliferation at 12 h, 24 h, and 48 h (*p* < 0.01, respectively), while 10 µM did not. Thus, 10 µM was chosen as a non-toxic concentration to study the effects of HIS on the IL-6/STAT3/S100A9 pathway, excluding direct tumor growth interference (Figure 5A).

To further explore the function of HIS in modulating the effects of stress hormones, A2780 and ES-2 cells were stimulated with the stress hormones NE and COR and then subsequently treated with HIS. We found that HIS treatment inhibited cell proliferation, migration, and invasion induced by stress hormones. Specifically, NE and COR treatment increased the number of colonies formed and promoted wound healing, cell migration, and invasion (*p* < 0.01, respectively). However, 10 µM HIS treatment decreased the number of colonies formed and promoted scratch healing, cell migration, and invasion (*p* < 0.01, respectively, Figures 5B–D).

### HDC regulated ovarian cell proliferation, migration, and invasion *in vitro*

**FIGURE 6:**
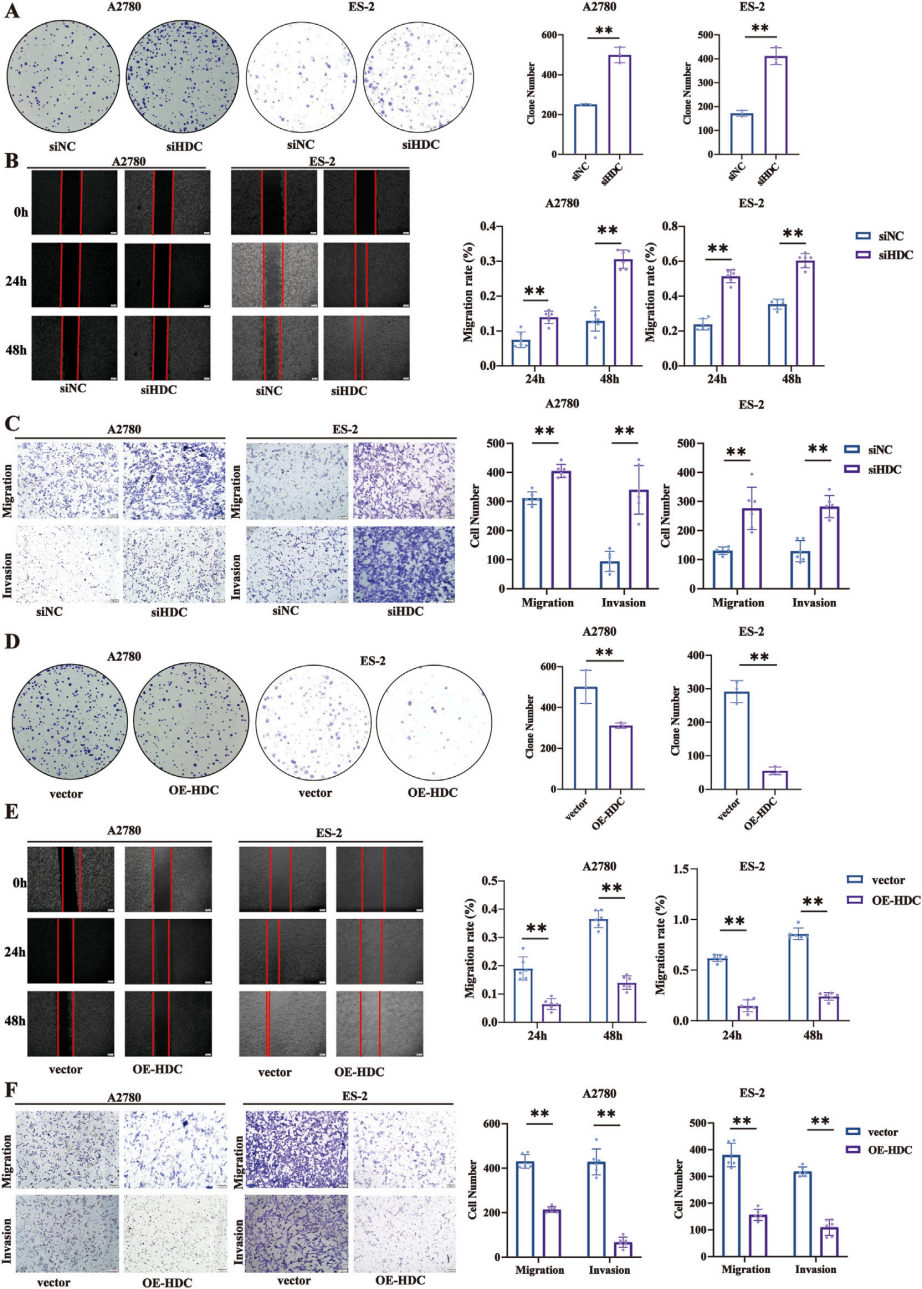
Knockdown/overexpression of HDC promoted/inhibited cell proliferation, migration, and invasion. **(A)** Colony formation assay in A2780 and ES-2 cells with knockdown of HDC. **(B)** Cell wound healing assay (100×) in A2780 and ES-2 cells with knockdown of HDC (n = 6 each). Scale bar = 200 μm. **(C)** Cell migration and invasion (100×) in A2780 and ES-2 cells with knockdown of HDC. Scale bar = 100 μm. **(D)** Colony formation assay in A2780 and ES-2 cells with overexpression of HDC (n = 3 each). **(E)** Cell wound healing assay (100×) in A2780 and ES-2 cells with overexpression of HDC. Scale bar = 200 μm. **(F)** Cell migration and invasion (100×) in A2780 and ES-2 cells with overexpression of HDC. Scale bar = 100 μm. Data are shown as mean ± SD. *p < 0.05; **p < 0.01.

Based on our previous results that chronic stress induced downregulation of HDC expression, we further explored the impact of HDC on tumor progression by constructing knockdown and overexpression of HDC in ovarian cell lines. Functionally, knockdown of HDC promoted cell proliferation, migration, and invasion in A2780 and ES-2 cells with siHDC compared with the NC groups (Figures 6A–C), while overexpression of HDC inhibited cell proliferation, migration, and invasion (Figures 6D–F).

### Stress hormones induced HDC expression downregulation, promoting ovarian cancer cell proliferation via the IL-6/STAT3/S100A9 pathway *in vitro*

In our preliminary experiments, we found that cells exposed to NE and/or COR downregulated the level of HDC protein and increased the phosphorylation of STAT3 and the expression of S100A9 (Supplementary Figure S2). The IL-6/STAT3/S100A9 pathway is a potential downstream target of HDC (Zhou et al., 2021). To elucidate the regulatory role of HDC in this signaling pathway, we conducted Western blot assay and ELISA to quantify protein expression and cytokine secretion in A2780 and ES-2 cell lines following various drug treatments, HDC knockdown, and HDC overexpression.

**FIGURE 7:**
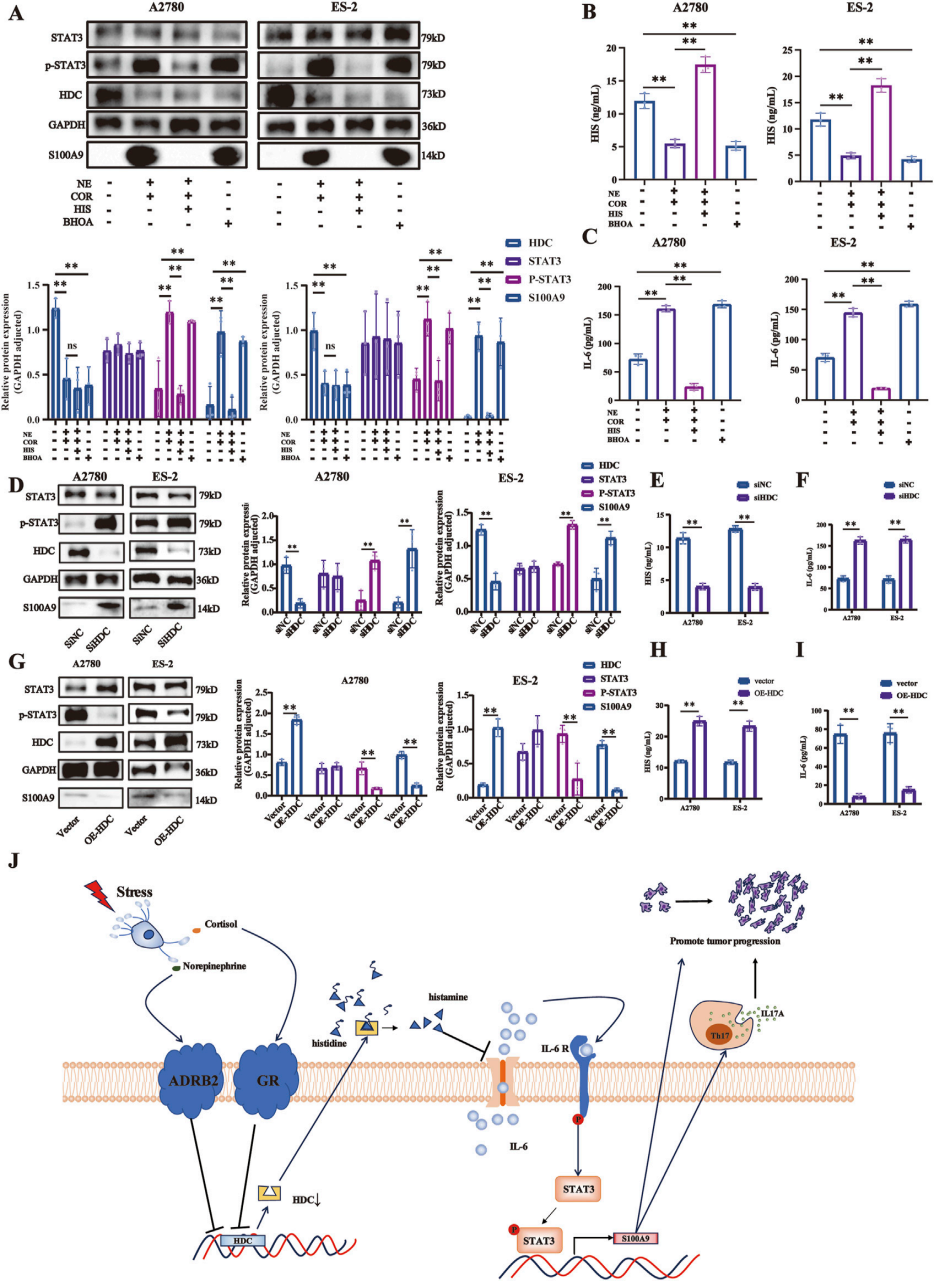
HDC downregulation induced by stress hormones promoted ovarian cancer cell proliferation via the IL-6/STAT3/S100A9 pathway. **(A)** Expressions of HDC, STAT3, p-STAT3, and S100A9 proteins in A2780 and ES-2 cells with various treatments of stress hormones (NE and COR), HIS, or HDC inhibitor (BHOA) detected by Western blot, adjusted by GAPDH. HIS **(B)** and IL-6 **(C)** levels in the cell supernatant of different groups in A2780 and ES-2 cells with various treatments detected by ELISA. **(D)** Expressions of HDC, STAT3, p-STAT3, and S100A9 proteins in A2780 and ES-2 cells with knockdown of HDC detected by Western blot, adjusted by GAPDH. HIS **(E)** and IL-6 **(F)** levels in the cell supernatants of different groups in A2780 and ES-2 cells with knockdown of HDC detected by ELISA. **(G)** Expressions of HDC, STAT3, p-STAT3, and S100A9 proteins in A2780 and ES-2 cells with overexpression of HDC detected by Western blot, adjusted by GAPDH. HIS **(H)** and IL-6 **(I)** levels in the cell supernatant of different groups in A2780 and ES-2 cells with overexpression of HDC detected by ELISA. **(J)** Mechanism diagram. Data are shown as mean ± SD. *p < 0.05; **p < 0.01.

Following various drug treatments, the IL-6/STAT3/S100A9 pathway expressions in A2780 and ES-2 cell lines were detected by Western blot (Figure 7A) and ELISA (Figures 7B, C). NE + COR decreased HDC but increased IL-6, p-STAT3 (Tyr 705), and S100A9 levels (*p* < 0.01, respectively), while HIS treatment induced the opposite changes, except for HDC. The HDC inhibitor BHOA decreased HDC but increased IL-6, p-STAT3 (Tyr 705), and S100A9 levels (*p* < 0.01, respectively).

Knockdown of HDC in A2780 and ES-2 cells reduced HIS but increased IL-6, p-STAT3, and S100A9 (*p* < 0.01, respectively), activating the IL-6/STAT3/S100A9 pathway (Figures 7D–F). Conversely, HDC overexpression increased HIS and reduced IL-6, p-STAT3, and S100A9 (*p* < 0.01, respectively), inhibiting the IL-6/STAT3/S100A9 pathway (Figures 7G–I).

Overall, these results indicated that HDC downregulation induced by stress hormones promoted the proliferation, migration, and invasion of ovarian cancer cells through activation of the IL-6/STAT3/S100A9 signaling pathway.

## Discussion

Chronic stress-induced ovarian cancer progression is a complex multistep process that requires the regulation of diverse cellular pathways and functions, which resulted in few studies on chronic stress that promotes ovarian cancer. In the present research, we revealed that HDC expression was downregulated by chronic stress in *in vivo* and *in vitro* experiments. The downregulation of HDC expression led to the activation of the IL-6/STAT3/S100A9 pathway, stimulating Th17 cells to secrete IL-17A, thereby promoting ovarian cancer progression, which suggests that HDC may be a potential therapeutic target for the comorbidity of ovarian cancer and depression.

Although many studies over the past several years have demonstrated that chronic stress and multiple genetic alterations of tumors can promote progression and metastasis of many cancers, such as breast, lung, gastric, and colorectal cancer (He et al., 2024; Zhang et al., 2019; Ye et al., 2023; Cao et al., 2024), the contribution of this information to the improvement of therapy is still small. In this research, we noted that CUMS promoted tumor growth in a successfully established comorbidity of ovarian cancer and depression *in vivo* mouse model, consistent with previous studies (Thaker et al., 2006), and provided a novel therapeutic target.

The HDC, HIS, and histaminergic system had been widely discussed in different neoplasms, including gastric, colorectal, esophageal, oral, pancreatic, liver, lung, skin, blood, and breast cancer (Massari et al., 2020). Some research studies hold opposing views to ours, considering HDC to be an oncogene to drive pro-angiogenic tumor microenvironment remodeling (Chen J. et al., 2022) or to enhance autocrine loop (Francis et al., 2012). However, an increasing number of research studies have suggested that HDC may function as a tumor suppressor gene to increase myeloid maturation and accumulation of CD11b+Ly6G + immature myeloid cells (Yang et al., 2011) to inhibit the growth of myc-dependent dedifferentiation tumors (Froldi et al., 2019). It has also been shown to inhibit myeloid maturation and CD8^+^ activation (Ahn et al., 2015). High HDC expression might predict a better clinical outcome, reducing the risk of cancer relapse, and might be a novel prognostic marker for breast cancer progression (Nicoud et al., 2020). Yet few studies have examined the role of HDC in ovarian cancer. Our findings have successfully established a connection between chronic stress and HDC, further strengthening the hypothesis that HDC expression downregulation contributes to cancer development.

However, few studies have examined the role of HDC in ovarian cancer. A previous study had shown that chronic stress promotes tumor progression through the activation of the β-adrenergic receptor-mediated cAMP-PKA signaling pathway, which enhances tumor angiogenesis in ovarian cancer (Thaker et al., 2006). In this study, we provide a novel mechanism by which chronic stress enhances ovarian cancer progression: chronic stress-induced HDC downregulation and alterations in histidine metabolism serve as critical factors regulating cancer progression. We confirmed this via IHC, Western blot, and qRT-PCR on tumor tissues. Additionally, HIS treatment counteracted CUMS-induced tumor growth, highlighting the importance of HDC. This study broadens the understanding of the role of depression in tumor progression, especially in ovarian cancer.

Moreover, HIS treatment reversed the elevated serum levels of inflammatory factors (IL-6 and IL-17A), stress hormones (NE and COR), and 5-HT, all of which have been shown to be upregulated under chronic stress and promote tumor progression, consistent with previous research (Gao et al., 2018; Heidt et al., 2014; Chen Y. et al., 2022; Ren et al., 2018). For example, chronic stress is known to trigger inflammatory responses, disrupt gut microbiota, activate IL-6/STAT3 signaling, and enhance immune responses, including DSS-induced colitis (Gao et al., 2018). Moreover, chronic stress elevates IL-6 production in rheumatoid arthritis (RA), reducing anti-inflammatory effects of glucocorticoids in RA patients (Davis et al., 2008), and promotes breast cancer metastasis by accumulating myeloid-derived suppressor cells via the activation of β-adrenergic signaling and the IL-6/STAT3 pathway (An et al., 2021). Notably, HIS treatment reversed the CUMS-induced upregulation of 5-HT, which was proven to inhibit tumor growth and enhance immune checkpoint blockade therapy (Schneider et al., 2021). While antidepressants such as fluoxetine and ketamine have been found to alleviate depressive symptoms and inhibit tumor growth (Yang et al., 2021; Ru et al., 2022; Schneider et al., 2021), few non-antidepressant medications have exhibited both anti-depression and anti-tumor therapeutic effects. This research first reported that HIS alleviated depression-like behaviors exacerbated by CUMS. HIS binds to different HIS receptors to exert various effects. It can not only secrete effector molecules such as IL-2 and IFN-γ but also activate cytotoxic effector cells such as NK cells and CD8^+^ T cells, thereby exerting anti-tumor activity. Simultaneously, it can also act on myeloid-derived suppressor cells (MDSCs) and promote the secretion of immunosuppressive cytokines such as IL-10 and IL-4, inducing the polarization of Th2 cells, inhibiting anti-tumor immunity, and promoting the occurrence and development of tumors (Nguyen and Cho, 2021). Previous research reported that 5 mg/kg HIS administration significantly decreased the proliferative potential of tumors and increased the apoptotic cell death and infiltration of lymphocytes (Nicoud et al., 2020). This study underscores the anti-inflammatory effects of HIS, in alleviating depression-like behaviors, and potentially enhancing immune checkpoint blockade therapy. These findings provide both theoretical and experimental evidence for the use of HIS in the treatment of depression comorbid with ovarian cancer.

Functional investigations indicate ovarian cancer progression via the IL-6/STAT3/S100A9 pathway. That IL-6 induces S100A9 expression in colonic epithelial cells through STAT3 activation has already been reported in recent studies about acute myeloid leukemia (AML), biliary tract cancer, and psoriasis-like skin and joint disease (Böttcher et al., 2022; Mellor et al., 2022; Ware et al., 2020). This research confirmed that chronic stress downregulated HDC expression in ovarian tumor models, upregulating the expressions of IL-6, p-STAT3, and S100A9. HIS reversed these changes, inhibiting stress hormone-induced wound healing, migration, and invasion. Furthermore, knockdown or overexpression of HDC activated or inhibited the expressions of IL-6, p-STAT3, and S100A9, thus supporting the finding that HDC, as an upstream cancer suppressor gene, catalyzes the formation of histamine, inhibiting ovarian cancer cell proliferation, migration, and invasion via the IL-6/STAT3/S100A9 pathway.

Furthermore, studies reported that chronic stress negatively affects the immune system and promotes tumor progression by mobilizing splenic myeloid cells and disrupting the M1–M2 polarization balance of tumor-associated macrophages through β-adrenergic signaling (Jiang et al., 2019; Yang et al., 2024). Notably, the IL-6/STAT3 pathway may regulate the differentiation of naive CD4^+^ T cells into Th17 cells (Zhao et al., 2018; Yoshida et al., 2016). We attempted to explain the influence of CUMS and HIS treatment on tumor progression from the perspective of cellular immunity and found that HIS treatment increased the proportion of T and Tc cells in an ovarian cancer mouse model, which suggested that HIS inhibited tumor progression. Tumor inoculation decreased T-cell proportion in mice spleen, which was further deteriorated by exposure to CUMS, and patients with depression have reduced circulating T cell. Depression promoted the proliferation and activation of Th17 cells, thereby exacerbating the inflammatory response and producing the inflammatory cytokine IL-17A (Beurel et al., 2022). We detected that IL-17A increase was induced by tumor and CUMS, which was reversed by HIS treatment. These findings confirm that IL-17A, secreted by Th17 cells, can promote tumor growth through the IL-6/STAT3 signaling pathway, as previously described (Wang et al., 2009). Specifically, chronic stress downregulated HDC expression, reducing HIS production and triggering excessive IL-6 secretion in cancer cells. This activated STAT3 and S100A9, suppressing T-cell formation, promoting Tc differentiation, and enhancing IL-17A secretion. Further studies are needed to elucidate the CUMS–cellular immunity–tumor interplay.

In conclusion, this study provides important insights into the effects of chronic stress on animal behavior, physiology, cellular functions, and immune system, as well as the underlying molecular mechanisms. Specifically, chronic stress elevates stress hormone levels, stimulates relative receptors, and subsequently decreases HDC expression, promoting ovarian cancer progression via the IL-6/STAT3/S100A9 pathway. A mechanism diagram is presented in Figure 7J. Histamine therapy may serve as a potential strategy to counteract the downregulation of HDC expression induced by chronic stress, providing crucial insights and establishing a theoretical foundation for further research on the treatment of ovarian cancer comorbid with depression.

## Data Availability

The original contributions presented in the study are included in the article/Supplementary Material; further inquiries can be directed to the corresponding authors.
