# Supplementary figures and images for: HDC downregulation induced by chronic stress promotes ovarian cancer progression via the IL-6/STAT3/S100A9 pathway
Source: Front Pharmacol. 2024 Dec 10;15:1485885. doi: 10.3389/fphar.2024.1485885 (PMC11666360; doi:10.3389/fphar.2024.1485885)

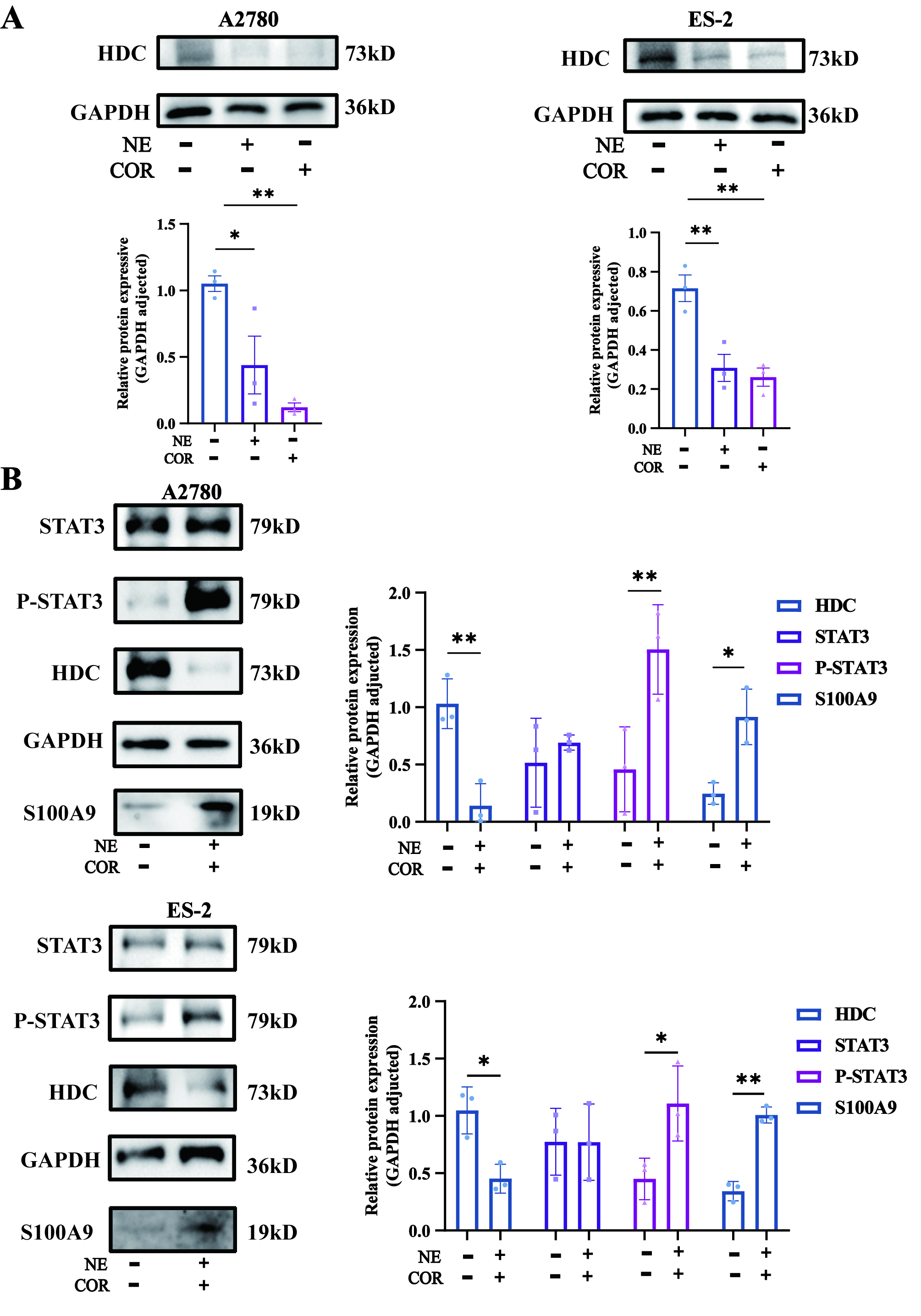

Supplement: Supplementary file 3 [file Image2.TIF]

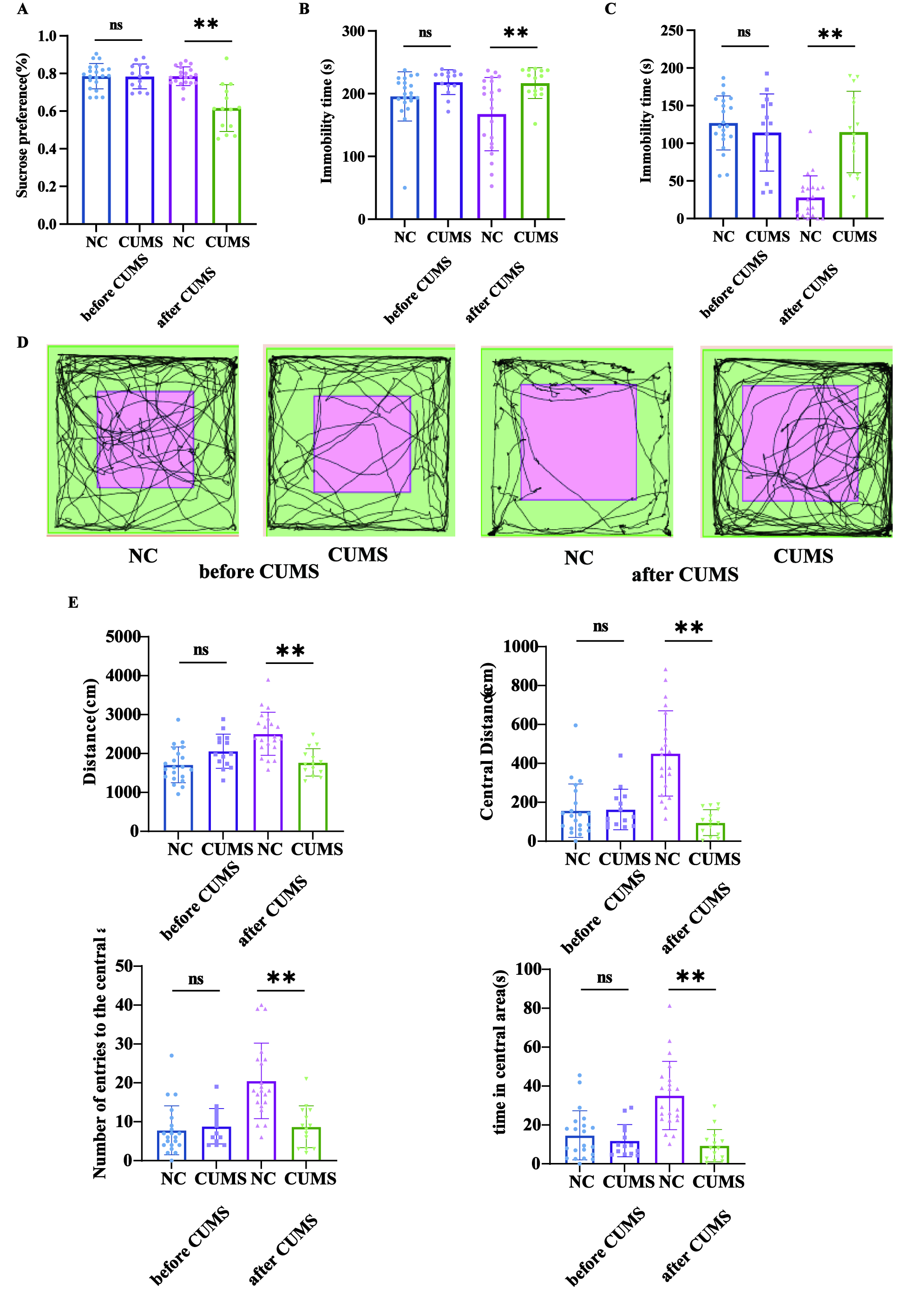

Supplement: Supplementary file 4 [file Image1.TIF]
